# Proteomic Insights into the Regulatory Mechanisms of the Freezing Response in the Alpine Subnivale Plant *Chorispora bungeana*

**DOI:** 10.3390/ijms252413381

**Published:** 2024-12-13

**Authors:** Hongyin Hu, Zhixing Zhao, Dongdi Ma, Lizhe An, Le Zhao, Xiule Yue

**Affiliations:** 1 Ministry of Education Key Laboratory of Cell Activities and Stress Adaptations, School of Life Sciences, Lanzhou University, Lanzhou 730000, China; huhy21@lzu.edu.cn (H.H.); zhaozhx2014@lzu.edu.cn (Z.Z.); 220220933570@lzu.edu.cn (D.M.); lizhean@lzu.edu.cn (L.A.); 2 State Key Laboratory of Efficient Production of Forest Resources, Beijing Forestry University, Beijing 100083, China; 3 School of Pharmacy, Henan University of Chinese Medicine, Zhengzhou 450046, China

**Keywords:** freezing stress, proteomic analysis, cold stress, differentially expressed proteins, *Chorispora bungeana*

## Abstract

Freezing temperatures impose significant constraints on plant growth and productivity. While cold tolerance mechanisms have been extensively studied in model species, the molecular basis of freezing tolerance in naturally adapted plants remains underexplored. *Chorispora bungeana*, an alpine plant with a strong freezing tolerance, provides a valuable model for investigating these adaptive mechanisms. In this study, we used Tandem Mass Tag (TMT)-based quantitative proteomics to analyze *C. bungeana* seedlings subjected to freezing stress (−6 °C) at 6 and 30 h, identifying 302 differentially expressed proteins (DEPs) compared with controls. Our findings capture the dynamic proteomic landscape of *C. bungeana* under freezing stress, revealing distinct early and prolonged responses. Early responses featured upregulated proteins involved in signaling and stress protection, with no clear involvement of the ICE1-CBF pathway (ICE1: Inducer of CBF Expression 1; CBF: C-repeat Binding Factor) found in cold-acclimating plants, while calcium signaling and epigenetic modifications enabled a rapid response. Extended exposure involved DEPs in RNA modification, glutamine metabolism, and biosynthesis of polysaccharides and flavonoids, highlighting metabolic adjustments crucial for long-term adaptation. By combining protein–protein interaction (PPI) networks and functional analysis, we identified 54 key proteins validated by qRT-PCR. These findings provide comprehensive insight into freezing tolerance mechanisms, identifying candidate proteins for enhancing cold resilience in crops and mitigating agricultural cold stress impacts.

## 1. Introduction

Cold stress, encompassing both chilling (0–15 °C) and freezing (<0 °C) temperatures, represents a significant abiotic stress that negatively impacts plant health and productivity by inhibiting seed germination, stunting growth, and reducing crop yield and quality [1,2,3]. These impacts arise as cold disrupts cellular processes, leading to dehydration and metabolic imbalance. Recent climate change has intensified temperature extremes worldwide, particularly increasing freezing events, which contribute substantially to economic losses in agriculture [4,5]. This highlights the urgent need to understand plant freezing tolerance mechanisms, which are critical for identifying freezing-resistant genes and improving crop resilience, thereby enhancing productivity and sustainability.

Over the past few decades, significant progress has been made in understanding plant responses to cold stress, revealing that plants utilize both rapid signaling and longer-term transcriptional adaptations [6,7,8]. Upon cold exposure, calcium ions (Ca^2+^) serve as critical secondary messengers, initiating downstream signaling pathways. In *Oryza sativa*, COLD1 (Cold Sensor 1) plays a pivotal role by modulating calcium influx in response to cold and interacting with G-protein signaling pathways [9]. This influx activates calcium-binding proteins, such as calcium-dependent protein kinases (CPKs), including CPK6 and CPK10 in *Arabidopsis thaliana*, which trigger cold-responsive pathways [10,11]. Reactive oxygen species (ROS) and plant hormones also mediate these responses [12,13]. Following cold perception, the ICE1-CBF pathway is activated, leading to the expression of cold-responsive (COR) genes such as *COR15A*, *COR47*, and *RD29A* [14,15]. These genes regulate metabolic adjustments, such as increasing the proportion of unsaturated fatty acids to maintain membrane fluidity [16,17], and accumulating osmoprotectants like proline and sugars (e.g., raffinose and trehalose) [18,19], ultimately helping plants mitigate the harmful effects of cold stress. However, most research on plant cold tolerance has focused on chilling stress, while mechanisms underlying freezing tolerance remain less explored.

While both chilling and freezing affect plants, the mechanisms underlying tolerance to each stress differ substantially. Chilling causes metabolic disruptions primarily due to enzyme inhibition at low temperatures. In contrast, freezing results in ice formation within cell walls, leading to cellular dehydration as water is extracted from within the cell [7]. Investigating the specific mechanisms of plant freezing tolerance will not only deepen our understanding of plant cold stress responses but also identify key genes for enhancing crop resilience to freezing temperatures.

*Chorispora bungeana* (Brassicaceae) is a perennial alpine herb highly adapted to freezing temperatures and frequent temperature fluctuations. Primarily found near Urumqi Glacier No. 1 in the Tianshan Mountains at altitudes above 3800 m, this species endures summer temperatures as low as 3–5 °C with intermittent snow and hail [20,21,22]. Given its resilience, *C. bungeana* serves as an excellent model for investigating plant freezing tolerance. Although several cold-responsive genes have been identified in this species, comprehensive omics-based studies to elucidate its freezing tolerance mechanisms and identify additional resistance genes are needed.

In this study, *C. bungeana* seedlings were subjected to freezing treatments at −6 °C for 6 and 30 h, followed by protein extraction. Using Tandem Mass Tag (TMT) technology, we analyzed differential protein expression under these conditions, identifying proteins associated with freezing tolerance by comparing them with untreated seedlings. GO and KEGG enrichment analyses were conducted, and representative key proteins were validated using qRT-PCR to assess their roles in freezing stress responses. Our findings offer new insights into cold stress adaptation and provide candidate genes for genetically enhancing crop freezing tolerance.

## 2. Results

### 2.1. Statistical Analysis of LC-MS/MS Results

**Figure 1:**
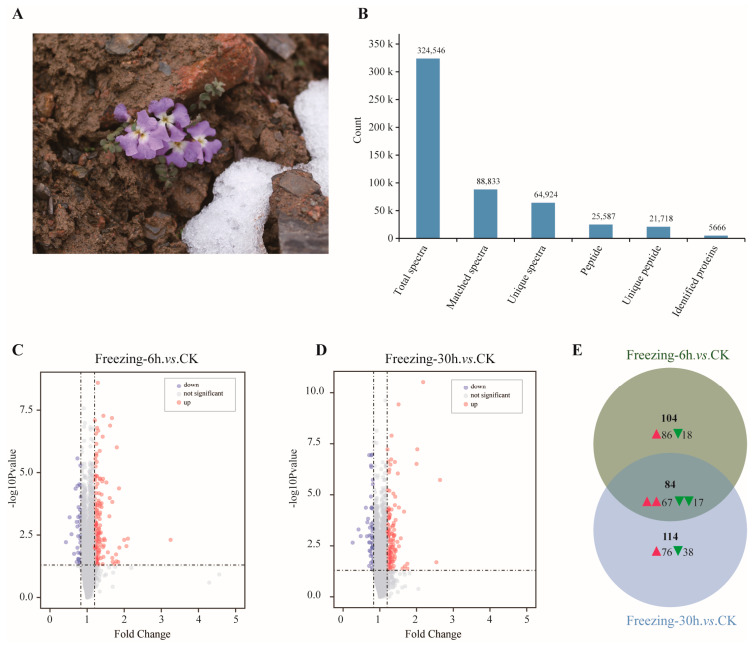
The basic statistical results of LC-MS/MS and DEP data. (**A**) The phenotypic characteristics and natural growth environment of *C. bungeana*. (**B**) The basic statistical information of MS data. (**C**) The volcano plot of DEPs in the ‘Freezing-6 h vs. CK’ comparison. The color gray represents proteins that are not differentially expressed in comparison with the control. The color red represents proteins that are upregulated, and the color blue represents downregulated proteins. (**D**) The volcano plot of DEPs in the ‘Freezing-30 h vs. CK’ comparison. (**E**) The Venn plot showed the DEPs in both comparisons and numbers of up- and downregulated DEPs. Red and green arrowheads represent up- and downregulated DEPs, respectively.

*Chorispora bungeana* is an alpine subnival perennial recognized for its adaptation to thrive in freezing environments (Figure 1A). To investigate the effect of freezing stress on the development of *C. bungeana*, proteomic analysis was performed on nine samples (each treatment combination consisting of three biological replicates) at three time points (0, 6, and 30 h). Following LC-MS/MS analysis, 324,546 spectra were acquired from *C. bungeana* under both control and freezing conditions, with 88,833 matching spectra identified. Of these, 25,587 peptides were validated by spectral analysis, including 21,718 unique peptides. As a result, 5666 proteins were identified and used for subsequent quantification (Figure 1B and Table S1). Additionally, analysis of the peptide length distribution showed that the majority consisted of 7-to-20 amino acids, consistent with quality control requirements (Figure S1). Further investigation assessed the statistical consistency of the quantitative results using the coefficient of variation (CV = SD/mean), with a CV of 15% typically considered satisfactory for method reproducibility. In this study, each sample had a mean CV of less than 10% (Figure S2), indicating a high level of statistical consistency in the protein quantification process for each sample.

### 2.2. Identification of DEPs Using TMT-Based Quantitative Proteomic Analysis

To investigate DEPs in response to freezing stress, the above-mentioned proteome was analyzed at multiple time points following freezing treatment using TMT quantitative proteomics. Among these proteins with notable changes in abundance (*p*-value < 0.05), those with fold changes > 1.2 or fold changes < 0.83 were identified as DEPs across the comparison groups (Figure 1C,D). The number of DEPs showing up- or downregulation between the different comparison groups is shown in Figure S3. The comprehensive statistical analysis results are shown in Table S2.

To gain a deeper insight into the proteomes of *C. bungeana* under freezing stress, samples from two key stress stages (Freezing-6 h and Freezing-30 h) were selected. We constructed two comparison groups: ‘Freezing-6 h vs. CK’ and ‘Freezing-30 h vs. CK’. In the comparison groups ‘Freezing-6 h vs. CK’ and ‘Freezing-30 h vs. CK’, we identified 188 and 198 significant DEPs, respectively (Figure 1E). Within the ‘Freezing-6 h vs. CK’ comparison, 153 proteins were upregulated and 35 were downregulated. Similarly, the ‘Freezing-30 h vs. CK’ comparison showed 143 upregulated and 55 downregulated proteins (Figure S4). Notably, 84 DEPs were common to both groups, suggesting that they are specifically induced by freezing stress, regardless of freezing treatment duration.

### 2.3. Subcellular Location and COGs Functional Classification of DEPs

**Figure 2:**
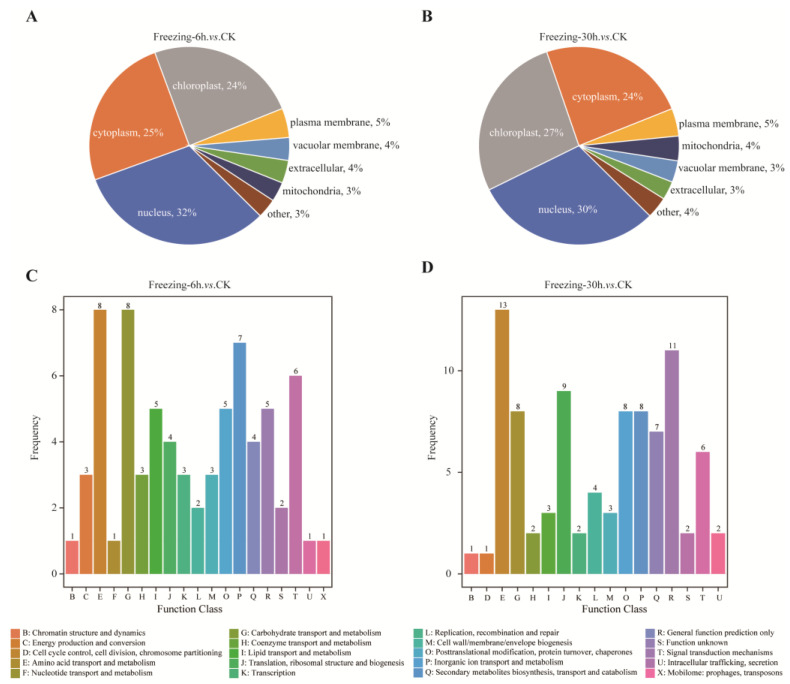
The statistical results of subcellular localization and COGs functional categories of DEPs. (**A**) Subcellular localization of DEPs in the ‘Freezing-6 h vs. CK’ comparison. The percentage represents the ratio of DEPs located in a specific subcellular structure. (**B**) Subcellular localization of DEPs in the ‘Freezing-30 h vs. CK’ comparison. (**C**) The COGs functional categories of DEPs in the ‘Freezing-6 h vs. CK’ comparison. (**D**) The COGs functional categories of DEPs in the ‘Freezing-30 h vs. CK’ comparison.

The putative subcellular localization of DEPs was predicted using the WoLF PSORT database. The analysis revealed that the majority of DEPs from both comparison groups (Freezing-6 h vs. CK and Freezing-30 h vs. CK) were predominantly localized in the chloroplast, cytoplasm, and nucleus, accounting for more than 81% in total (Figure 2A,B). In addition, DEPs were categorized into different functional groups based on the COGs database alignment in different comparison groups. In the ‘Freezing-6 h vs. CK’ comparison, the predominant COGs categories were ‘amino acid transport and metabolism’, ‘carbohydrate transport and metabolism’, and ‘inorganic ion transport and metabolism’ (Figure 2C). Conversely, in the ‘Freezing-30 h vs. CK’ comparison, the top three COGs categories were ‘amino acid transport and metabolism’, ‘general function prediction only’, and ‘translation, ribosomal structure and biogenesis’ (Figure 2D). This result indicates that the DEPs had a different regulation of functional groups in response to freezing stress at different time points.

### 2.4. GO and KEGG Enrichment of DEPs in Freezing Stress

**Figure 3:**
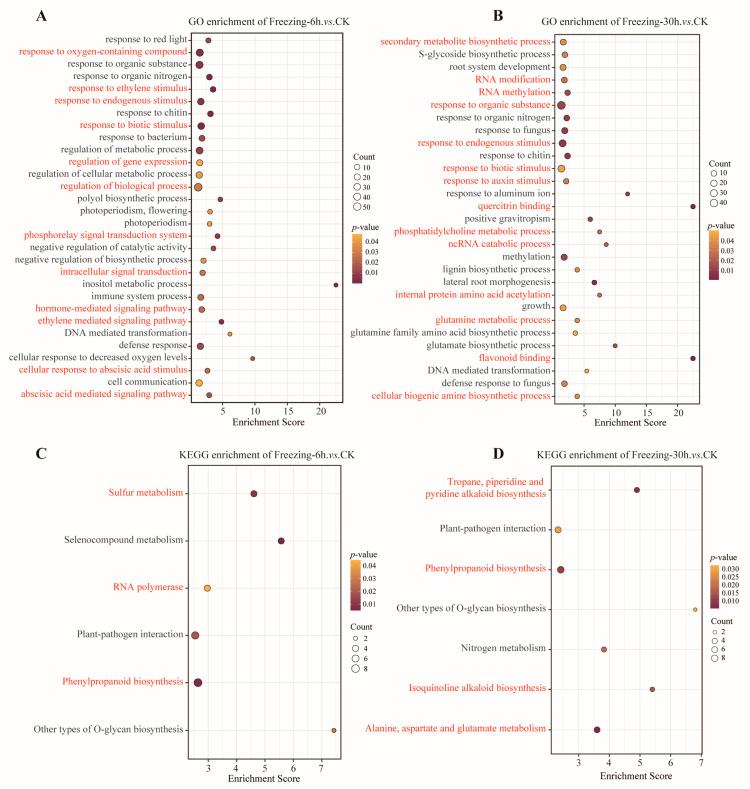
The enrichment analysis of DEPs in different comparisons. (**A**) GO enrichment analysis of DEPs in the ‘Freezing-6 h vs. CK’ comparison. The red terms represent functions that may be of significant importance that are related to the freezing response in *C. bungeana*. (**B**) GO enrichment analysis of DEPs in the ‘Freezing-30 h vs. CK’ comparison. (**C**) KEGG pathways enrichment analysis of DEPs in the ‘Freezing-6 h vs. CK’ comparison. (**D**) KEGG pathways enrichment analysis of DEPs in the ‘Freezing-30 h vs. CK’ comparison.

To elucidate the biological function of freezing stress-induced DEPs in *C. bungeana*, we annotated these proteins with GO terms and performed enrichment-based clustering analysis using three ontologies: biological process (BP), cellular component (CC), and molecular function (MF). In the ‘Freezing-6 h vs. CK’ comparison, DEPs showed significant enrichment in functions such as ‘response to ethylene stimulus’, ‘phosphorelay signal transduction system’, ‘defense response’, and ‘abscisic acid-mediated signaling pathway’ (Figure 3A and Table S3). Similarly, for the DEPs in the ‘Freezing-30 h vs. CK’ comparison, the enriched functions included the ‘secondary metabolite biosynthetic process’, ‘RNA modification’, ‘glutamine metabolism process’, and ‘flavonoid binding’ (Figure 3B and Table S4). Some phytohormones, including abscisic acid and ethylene, have been identified as pivotal signaling mediators in the regulation of the freezing response. Furthermore, certain secondary metabolites, including polysaccharides, flavonoids, and alkaloids, serve as direct protective agents against freezing stress in plants. These results suggest that the DEPs involved in these processes may play key roles in the freezing response.

To gain further insight into the function of the DEPs from a pathway-specific standpoint, we also conducted a KEGG pathway enrichment-based clustering analysis. In the comparison between ‘Freezing-6 h vs. CK’, the DEPs were linked to the pathways of ‘Sulfur metabolism’, ‘RNA polymerase’, and ‘Phenylpropanoid biosynthesis’ (Figure 3C). In contrast, the DEPs demonstrated notable correlations with the pathways of ‘Tropane, piperidine and pyridine alkaloid biosynthesis’, ‘Isoquinoline alkaloid biosynthesis’, and ‘Alanine, aspartate and glutamate metabolism’ in the ‘Freezing-30 h vs. CK’ comparison (Figure 3D). Sulfur and sulfur-containing compounds act as signaling molecules that regulate the synthesis of secondary metabolites, which are of great importance for enhancing plant resistance against oxidative stress and pathogenicity. Alkaloid biosynthesis plays a significant role in plant freezing resistance, including growth and development, antioxidant defense, and signal transduction.

### 2.5. Protein–Protein Interaction (PPI) Analysis of DEPs Regulated by Freezing Stress

**Figure 4:**
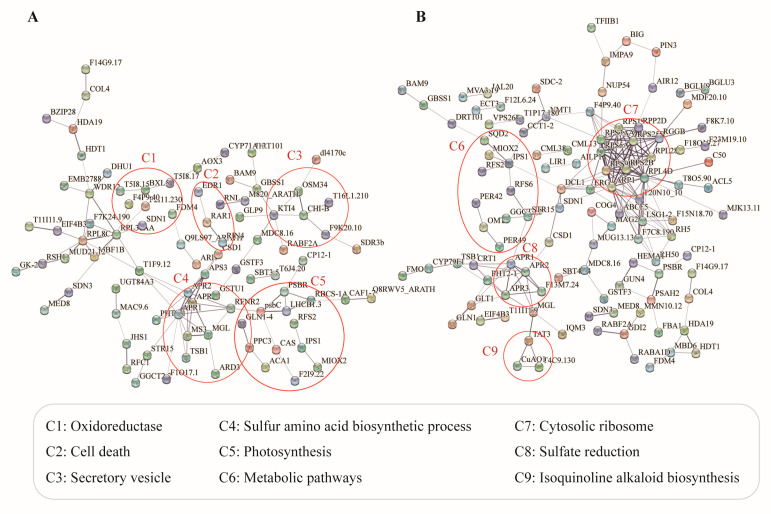
Protein-protein interaction networks of DEPs from TMT-based proteomics. (**A**) Interaction network of DEPs in the ‘Freezing-6 h vs. CK’ comparison. (**B**) Interaction network of DEPs in the ‘Freezing-30 h vs. CK’ comparison.

To gain a deeper understanding of the regulatory mechanisms of DEPs and their roles in the response to freezing stress, the DEPs with the most significant interaction relationships were selected from the ‘Freezing-6 h vs. CK’ and ‘Freezing-30 h vs. CK’ comparisons based on the STRING database (Tables S5 and S6). In the comparison of ‘Freezing-6 h vs. CK’, the DEPs were observed to interact with each other and were involved in several processes, including ‘Sulfur amino acid biosynthetic process’, ‘Photosynthesis’, ‘Oxidoreductase’, and ‘Secretory vesicle’ (Figure 4A). In the PPI networks of the ‘Freezing-30 h vs. CK’ comparison, the annotated DEPs were associated with functions including ‘Cytosolic ribosome’, ‘Metabolic pathways’, ‘Sulfate reduction’, and ‘Isoquinoline alkaloid biosynthesis’ (Figure 4B). For instance, the adenosine 5′-phosphosulfate reductases (APR1, APR2, and APR3), which are involved in primary sulfate reduction, are also activated by the MYB factors in response to cold stress [23]. Additionally, the Calcium Sensor (CAS) protein has been reported to play a crucial role in calcium signal transduction, mediating the plant’s response to cold stress [24].

**Figure 5:**
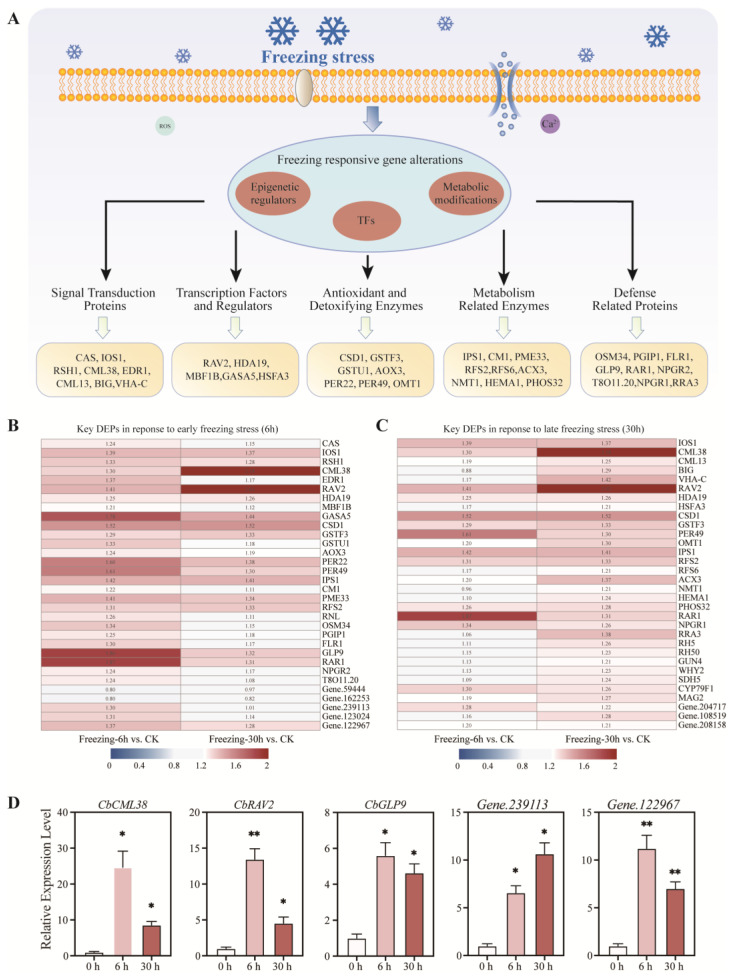
Key proteins’ expression and qRT-PCR verification. (**A**) The overview of key proteins’ function in the freezing response. (**B**) The heatmap of key DEPs’ quantification in the ‘Freezing-6 h vs. CK’ comparison. (**C**) The heatmap of key DEPs’ quantification in the ‘Freezing-30 h vs. CK’ comparison. (**D**) Transcriptional expression of five DEPs in freezing treatments of *C. bungeana*. Error bars indicate SD (*n* = 5). Statistical significance was determined using Student’s *t*-test, with asterisks indicating significant correlations (* *p* < 0.05, ** *p* < 0.01 compared with 0 h).

In light of the preceding bioinformatics analysis and protein function, a total of 54 critical DEPs were selected and are presented in Table S7. These proteins are involved in several different functions, including ‘signal transduction’, ‘transcription factors and regulators, ‘antioxidant and detoxifying enzymes’, ‘metabolism-related enzymes’, and ‘defense-related proteins (see Figure 5A and Table S8 for details). Of the key DEPs, 30 were found to be upregulated in the ‘Freezing-6 h vs. CK’ comparison (Figure 5B and Table S9), while 32 were unregulated in the ‘Freezing-30 h vs. CK’ comparison (Figure 5C and Table S10). Furthermore, eight DEPs were identified as novel proteins not previously annotated in any public databases. The domains of these novel proteins were then predicted and depicted in Figure S5. To evaluate the accuracy of the TMT-based quantitative proteomics analysis, five genes were selected for quantitative real-time PCR (qRT-PCR) analysis (Table S11). The expression of these genes was found to undergo significant alterations in comparison to the control group when subjected to freezing stress. The overall expression patterns of the selected genes were consistent, further validating the reliability of the TMT-based proteomics results (Figure 5D).

## 3. Discussion

### 3.1. Chorispora bungeana as a Model for Investigating Freezing Tolerance Research

*Chorispora bungeana,* an alpine plant native to the subnival zones of the Tianshan Mountains, thrives even under snow cover (Figure 1A), making it an ideal model for studying freezing tolerance mechanisms in plants. Over the past decades, we have developed a reliable regeneration and cultivation system for *C. bungeana* seedlings, enabling year-round research independent of seasonal and environmental factors. This system ensures a consistent supply of seedlings, essential for in-depth investigations into freezing tolerance. In addition, our ongoing T2T genomic sequencing and comparative genomic studies are enhancing our understanding of *C. bungeana*’s genetic framework, supporting future research on cold tolerance mechanisms.

Our proteomic analysis, using LC-MS/MS and TMT-based quantitative proteomics, identified 5666 proteins in *C. bungeana* exposed to freezing stress at 0, 6, and 30 h. Notably, 188 and 198 DEPs were detected at 6 and 30 h, respectively, with 84 DEPs common to both time points. These proteins are involved in critical processes such as amino acid metabolism, carbohydrate transport, ion homeostasis, and stress response, reflecting the plant’s metabolic reprogramming under freezing conditions. Additionally, proteins related to phytohormone-regulated signaling, particularly those associated with abscisic acid (ABA) and ethylene, were upregulated, suggesting that *C. bungeana* coordinates its freezing response through hormonal regulation [20,25,26]. Furthermore, the analysis revealed an increase in proteins involved in secondary metabolite biosynthesis, including flavonoids and alkaloids. These metabolites play a crucial role in mitigating oxidative damage by scavenging reactive oxygen species (ROS), with enriched pathways related to phenylpropanoid and alkaloid biosynthesis supporting their protective function in freezing tolerance [27,28].

Together with our tissue culture system and genomic research, these proteomic findings provide a robust foundation for advancing the study of freezing tolerance. By integrating these complementary approaches, *C. bungeana* emerges as a promising model for understanding the molecular basis of cold tolerance, offering valuable insights to drive both fundamental and applied research aimed at improving freezing resistance in plants.

### 3.2. Early Freezing Response of C. bungeana, Lacking ICE1 and CBFs in Freezing Conditions

In *C. bungeana*, early responses to freezing stress involve the upregulation of proteins related to signaling and stress protection rather than the classic ICE1-CBF pathway observed in many cold-acclimating plants [7]. While the ICE1-CBF-COR pathway is a primary mechanism for initiating cold responses in plants like *Arabidopsis thaliana*, the absence of ICE1 and CBFs at the 6 h mark in *C. bungeana* may suggest a unique response strategy. It is possible that ICE1 and CBF proteins act very early in response to cold, initiating downstream cold-responsive genes and rapidly decreasing afterward, making them undetectable in TMT proteomics at this later stage. Evidence from other studies supports this dynamic response, where ICE1 and CBF proteins peak shortly after cold exposure and then diminish as downstream genes become active [6,29].

The upregulation of calcium-binding proteins (e.g., CML38 and CAS) and receptor-like kinases (IOS1) during the early freezing response suggests that calcium signaling plays a critical role in *C. bungeana*’s response to initial cold shock. Calcium ions function as secondary messengers, activating proteins that modulate stress responses, and are crucial for initiating protective mechanisms under freezing conditions [29]. Additionally, the upregulation of ROS-scavenging enzymes (e.g., Superoxide Dismutase CSD1, Peroxidases PER22, PER49, and Glutathione S-Transferase GSTF3) indicates an early response to mitigate oxidative damage. ROS production is a common stress response, but without adequate scavenging ROS can damage cellular components, underscoring the importance of these enzymes in maintaining cell integrity during freezing [30,31].

Histone Deacetylase 19 (HDA19) appears in the early response phase, pointing to an epigenetic component that may influence stress-responsive gene expression. Epigenetic modifications, such as histone deacetylation, could provide rapid adaptability to environmental changes, a mechanism that aligns with findings in other plant species where chromatin remodeling is part of cold acclimation [31,32].

### 3.3. Late Freezing Response of C. bungeana

By the 30 h mark, *C. bungeana* shifts its response to a set of proteins that support sustained tolerance rather than immediate stress mitigation. The upregulation of Auxin Transport Protein BIG and V-type Proton ATPase Subunit C (VHA-C) suggests adjustments in cellular pH and ion transport, which are critical for maintaining cell stability under prolonged stress. These proteins are typically associated with ion homeostasis, a function vital to preventing ion imbalance and pH shifts, which can exacerbate stress damage if left unregulated [33,34].

The upregulation of Heat Stress Transcription Factor A-3 (HSFA3) highlights a connection between heat and cold stress responses, as HSFA3 is involved in stabilizing proteins and preventing denaturation under stress [35,36]. The presence of HSFA3 may suggest that cold-tolerant plants like *C. bungeana* can leverage cross-protection mechanisms typically associated with heat stress, which can contribute to structural protein stability under freezing conditions [33].

Additional proteins, such as RNA helicases (RH5 and RH50) and Tetrapyrrole-Binding Protein (GUN4), support transcription and photosynthetic function, respectively, under stress conditions [37]. These proteins are vital for maintaining metabolic activity during freezing, which is essential for long-term stress adaptation and energy balance. Their presence aligns with findings in other cold-tolerant species, where energy metabolism remains active even in adverse conditions [6].

### 3.4. Possible Roles of Uncharacterized Proteins in Freezing Stress

The uncharacterized proteins identified in *C. bungeana* during freezing stress likely play key roles in membrane stabilization, osmotic regulation, and signaling under extreme cold. In freezing-tolerant species like rye, novel ice-binding proteins (IBPs) help maintain cellular integrity by stabilizing membrane structures and controlling ice formation, suggesting that these uncharacterized proteins in *C. bungeana* may perform similar protective functions [31]. These proteins could also act as cryoprotectants, synthesizing or modulating unique osmolytes to prevent cellular dehydration during ice crystallization. Such mechanisms are crucial for high-altitude species like *C. bungeana*, where cryoprotective adaptations enable survival in extreme conditions [29,31]. In addition, uncharacterized proteins may be involved in alternative regulatory pathways distinct from the ICE1-CBF system, enabling flexible and rapid responses to freezing through novel signaling components. Further functional studies on these proteins may reveal new targets for enhancing freezing tolerance in other plants [29].

## 4. Materials and Methods

### 4.1. Plant Materials and Freezing Treatments

*Chorispora bungeana* specimens were collected from the Tianshan Mountains in Xinjiang Province, China. Regenerated plants, developed according to methods detailed in previous research [20], were maintained in a growth chamber at 20 °C with a 16 h light/8 h dark cycle and subcultured every two weeks. Seven-day-old regenerated seedlings were used for the present study. To investigate freezing tolerance, the seedlings were first exposed to 0 °C for 0.5 h, and ice chips were sprinkled on them. The chamber temperature was then gradually decreased at a rate of −2 °C per hour, with the freezing treatment starting once the temperature reached −6 °C. Leaves from the seedlings were sampled after 6 and 30 h of freezing exposure, with ten seedlings per sample and three replicates for each time point. Leaves from non-treated seedlings (0 h), kept under normal conditions, served as controls (CK). Following freezing treatments, all samples were immediately collected, flash-frozen in liquid nitrogen, and stored at −80 °C for subsequent quantitative proteomic analysis.

### 4.2. Protein Extraction, Digestion, and TMT Labeling

The TMT-based quantitative proteomic analysis was performed by BGI Genomics Co., Ltd. in Shenzhen, China. The experimental samples were derived from three treatment combinations, each consisting of three biological replicates. The leaves were crushed using liquid nitrogen. Samples were treated with a 5× volume of 80% acetone and 10 mM DTT (DL-dithiothreitol), followed by three rounds of ultrasonication on ice. An equal volume of Tris-saturated phenol was then added to the mixture, which was then centrifuged at 4 °C and 10,000× g for 10 min. The supernatant was combined with a 5× volume of 0.1 M ammonium acetone-saturated methanol and 10 mM DTT and incubated at −20 °C for at least 12 h. After centrifugation, the precipitate was washed once with ice-freezing methanol, followed by three washes with ice-freezing acetone. The protein precipitate was then redissolved in Lysis Buffer 3, sonicated in an ice bath for 5 min, and centrifuged at 25,000× g (4 °C) for 15 min and the concentration was determined using a bicinchoninic acid assay kit according to the manufacturer’s guidelines. Protein quality was confirmed by sodium dodecyl sulphate–polyacrylamide gel electrophoresis (SDS-PAGE) with equal protein loading for all samples. Trypsin was added to the 100 μg protein solution samples at a 1:40 mass ratio (trypsin:protein) for the initial 4 h digestion, followed by an 8 h second digestion. After digestion, the peptides were desalted using a Strata X C18 column (Phenomenex, Torrance, CA, USA), followed by lyophilization under vacuum conditions. The solubilized peptides were then labeled using the TMT kit (Thermo, Waltham, MA, USA) according to the manufacturer’s instructions. The labeled peptide mixtures were then combined, desalted, and subjected to another round of vacuum freeze-drying.

### 4.3. HPLC Fractionation and LC-MS/MS Analysis

Peptides labeled with TMT were fractionated using high-pH reversed-phase high-performance liquid chromatography (HPLC) with a Shimadzu LC-20AD C18 column. In brief, the peptides were first separated into 60 fractions using a gradient of 8%-to-32% acetonitrile (pH 9.0) over 60 min. The peptides were then consolidated into nine fractions and subjected to vacuum freeze-drying. The fractions were reconstituted in 0.1% formic acid (Fluka, Seelze, Germany) and then applied to a reversed-phase analytical column using an EADY-nLC 1000 UPLC system (Thermo, Waltham, MA, USA) at a constant flow rate of 400 nL/min. The peptides were then subjected to nano-electrospray ionization (nano-ESI) followed by tandem mass spectrometry (MS/MS) using a Q Exactive Plus instrument (Thermo, Waltham, MA, USA) coupled online to the UPLC system. An electrospray voltage of 1.6 kV was used. MS scan spectra were acquired in the mass-to-charge (*m*/*z*) range of 350 to 1600, and intact peptides were detected in the Orbitrap at a resolution of 70,000. The MS/MS scan spectra were selected in the Orbitrap at a resolution of 17,500.

### 4.4. Protein Identification and Quantification

The MS/MS data obtained were analyzed and processed using the Mascot (v2.3.02) [38] search engine, which searches with the *C. bungeana* proteins derived from the PacBio full-length cDNA sequences (CNCB, PRJCA030740) to obtain the final protein identification result. A mass tolerance of 20 ppm was set for precursor ions in the initial search, followed by a mass tolerance of 5 ppm in the main search. A mass tolerance of 0.05 Da was used for fragment ions. Carbamidomethyl on cysteine was designated as a fixed modification, while oxidation on methionine was designated as a variable modification. TMT quantification data were processed using Iquant [39], a proprietary tool developed by BGI, with a false discovery rate (FDR) criterion set at >1%. Only proteins identified with at least one unique peptide were retained for further analysis. Proteins with a fold change >1.2 or <0.83, together with a *p*-value < 0.05, were considered to be differentially abundant proteins (DEPs).

### 4.5. Subcellular Localization and COGs Function Classification

To predict the subcellular localization of DEPs, the WoLF PSORT [40] tool (https://www.genscript.com/wolf-psort.html) (accessed on 21 September 2024) available at GenScript was used. For the functional classification of DEPs, the Cluster of Orthologous Groups of Proteins (COGs) database [41] from the National Center for Biotechnology Information (NCBI) was used. Protein alignment was performed using the Diamond Blastp program [42].

### 4.6. Annotation and Enrichment Analysis of the GO Function and KEGG Pathways

Function annotation was performed using the eggNOG-mapper [43] to predict the GO function and KEGG pathways of proteins based on protein sequence alignment. The enrichment of the GO function and KEGG pathways for identified proteins was analyzed using TBtools (v2.095) [44] with a two-tailed Fisher’s exact test. Enrichment was considered significant for the GO function or KEGG pathways with a corrected *p*-value < 0.05. Categories showing significant enrichment in at least one of the different comparison groups were selected and subjected to one-way hierarchical clustering.

### 4.7. PPI Network Analysis

Protein database accessions from different comparison groups were individually queried in the STRING database (https://www.string-db.org/) (accessed on 25 September 2024) to identify protein–protein interactions (PPI). A confidence score threshold of >0.4 (medium confidence) was used to construct the interaction network of DEPs. To improve visualization, the proteins with direct interaction relationships and the proteins directly linked to them were selected. The resulting network was visualized using Cytoscape (v3.7.2) software [45].

### 4.8. RNA Isolation and qRT-PCR Detection

The isolation of total RNA was performed using the RNAprep Pure Plant Kit (TIANGEN, Beijing, China). Single-stranded cDNA was synthesized with the Evo M-MLV RT Premix for qPCR Kit (AGbio, Changsha, China) following the manufacturer’s instructions. Gene-specific primers for quantitative real-time PCR (qRT-PCR) were designed using the software Primer Premier (v5.0) (PREMIER Biosoft International, San Francisco, CA, USA). The primers are provided in Table S11. The qRT-PCR analysis was conducted with SYBR Green Premix (AGbio, Changsha, China) under the manufacturer’s instructions on the ABI Q5 PCR Real-Time Thermal Cycler (Applied Biosystems, Foster City, CA, USA). Gene expression levels were determined using the Pfaffl method [46].

## 5. Conclusions

This study provides new insights into the mechanisms underlying freezing tolerance in *Chorispora bungeana*, a plant naturally adapted to extreme cold. Through TMT proteomics analysis, we identified a range of upregulated proteins during early (6 h) and late (30 h) freezing treatments at −6 °C. Early responses were characterized by rapid activation of calcium signaling, ROS-scavenging enzymes, and epigenetic regulators, whereas the late phase involved proteins supporting ion balance, sustained metabolic activity, and cross-protection typically associated with heat stress.

Furthermore, the presence of uncharacterized proteins points to potentially novel components in *C. bungeana*’s freezing tolerance strategy, likely contributing to membrane stability, osmotic regulation, and signaling. These findings reveal *C. bungeana* as a valuable model for understanding freezing tolerance and offer promising candidates for improving cold resilience in crops. Future research on these uncharacterized proteins could uncover unique pathways and molecular targets to enhance freezing tolerance in agricultural species.

## Data Availability

The spectral raw data and resolved peptides generated in this study have been submitted to the integrated Proteome Resources (iProX) database [47] under the accession number IPX0010099001.

## Supplementary Materials

The following supporting information can be downloaded at https://www.mdpi.com/article/10.3390/ijms252413381/s1.
