# Supplementary material for: Proteomic Insights into the Regulatory Mechanisms of the Freezing Response in the Alpine Subnivale Plant Chorispora bungeana
Source: Int J Mol Sci. 2024 Dec 13;25(24):13381. doi: 10.3390/ijms252413381 (PMC11678613; doi:10.3390/ijms252413381)
Supplement: Supplementary file 1 [file ijms-25-13381-s001.zip › Supplementary Figures-Proteomic analysis of Chorispora bungeana.pdf]

Supporting Information

Supplement Figure

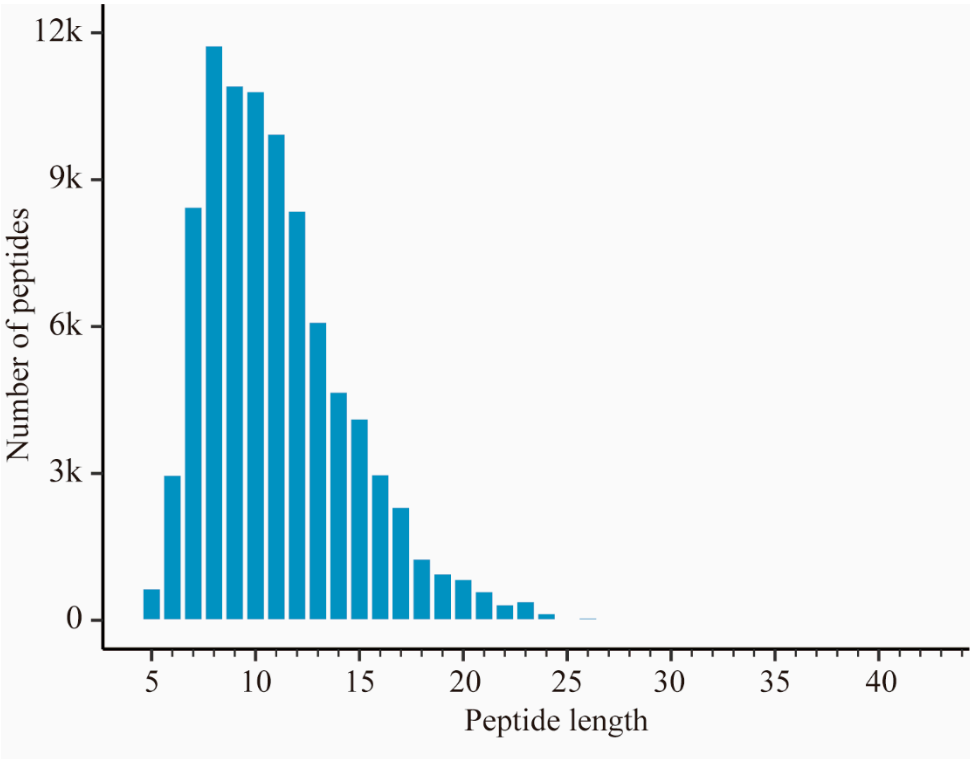

Figure S1. The length distribution of peptides.

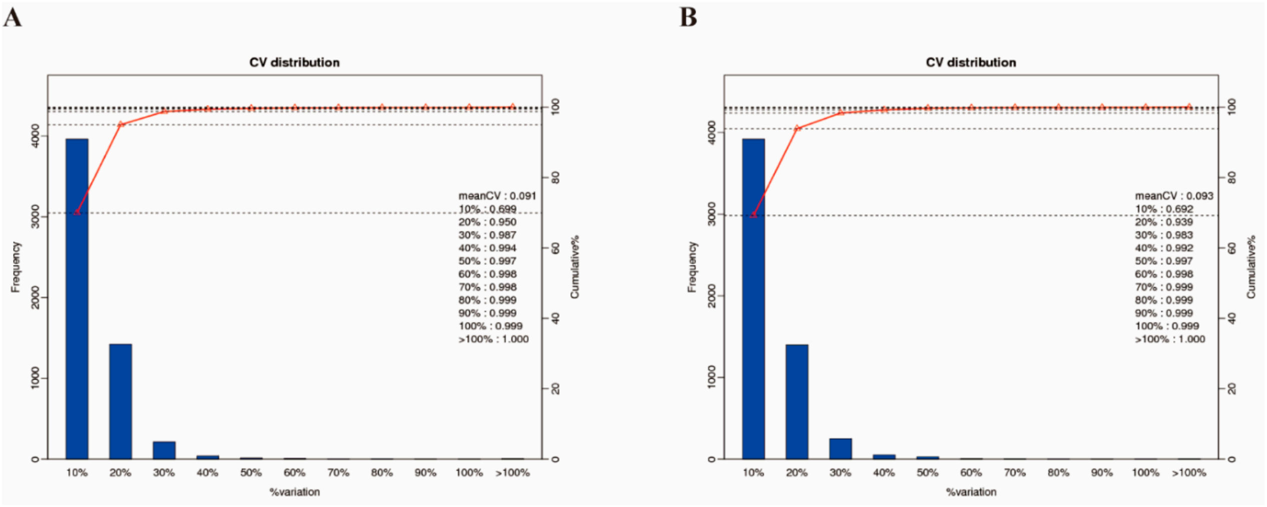

Figure S2. The CV value distribution of different samples in 6h (A) and 30 h freezing stress (B).

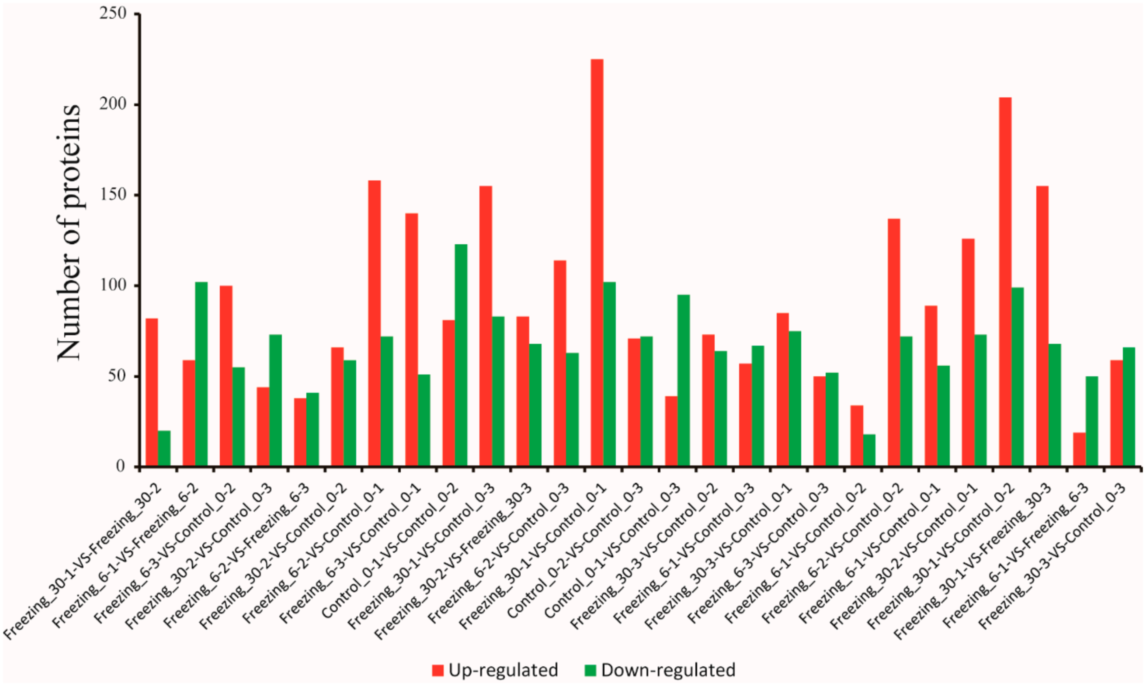

Figure S3. Numbers of up- and down-regulated DAPs among various comparisons.

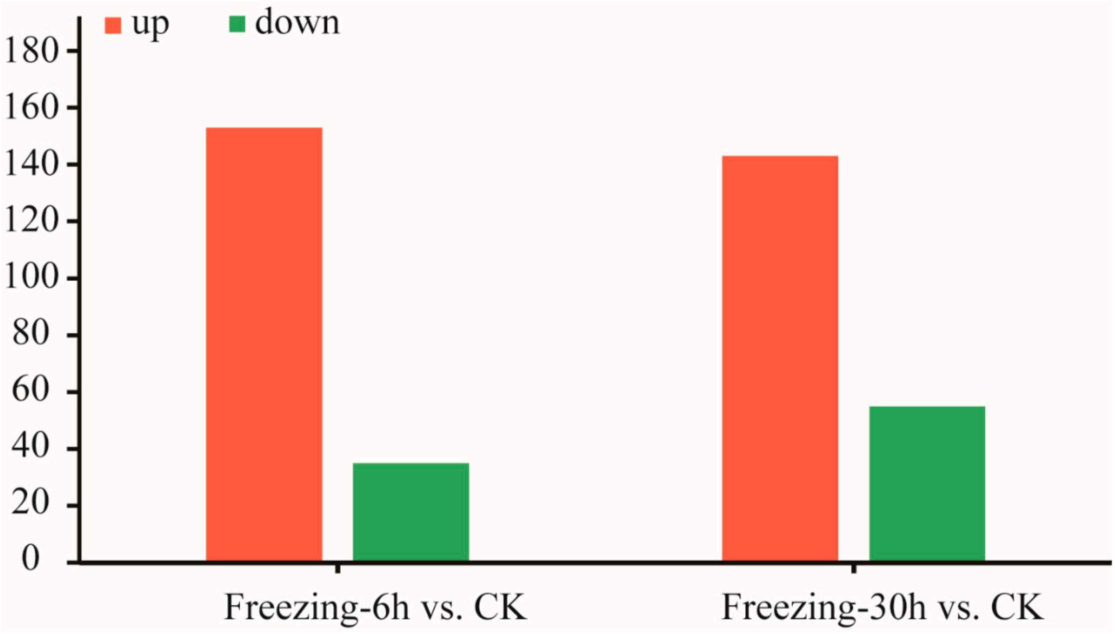

Figure S4. Numbers of up- and down-regulated DAPs of two groups of Freezing-6h vs. CK and Freezing-30h vs. CK.

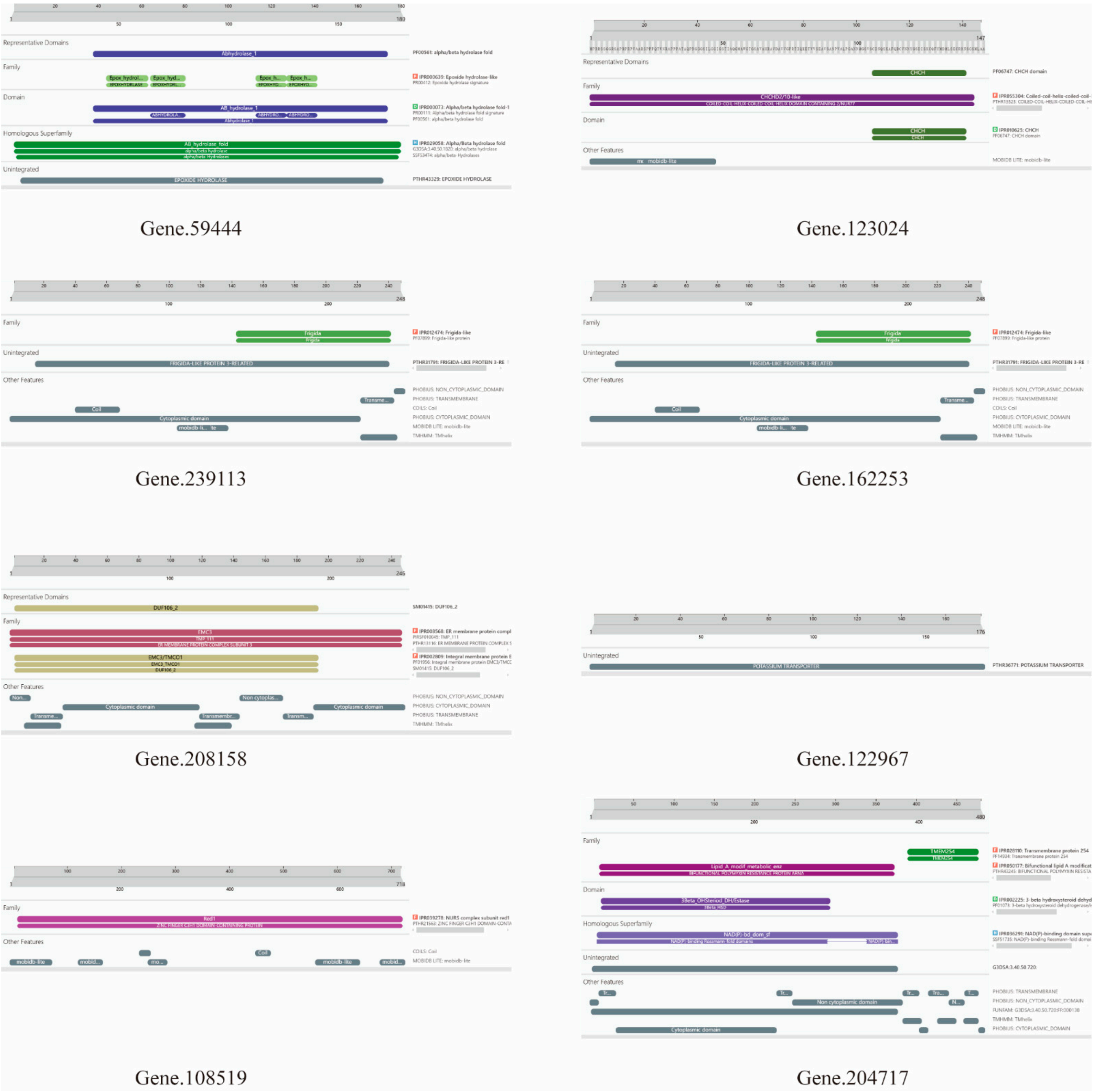

Figure S5. The domains predicted of novel proteins.
